# Quantum sensing of noises in one and two dimensional quantum walks

**DOI:** 10.1038/s41598-017-04795-2

**Published:** 2017-07-10

**Authors:** Tian Chen, Xiong Zhang, Xiangdong Zhang

**Affiliations:** School of Physics, Beijing Institute of Technology, Beijing, 100081 China

## Abstract

Quantum walk (QW) provides a versatile platform for the realization of quantum algorithms. Due to the existence of the inevitable noises in the walk, the different quantum algorithms accommodating to different noises are demanded. Thus, the success of the algorithms based on the QW requires us to sense different noises in the walk. Until now, the way to distinguish different noises in the walk has been discussed rarely. Here, we propose an efficient way to sense the noises in the one and two dimensional QWs. The populations of the coin in the walk with or without decoherence are presented. By only detecting the populations of the coin in the QW, we can determine whether there exists the decoherence in the total QW system. Moreover, the non-Markovianity of the coin in the one and two dimensional QWs is revealed, in which the coin is taken as an open quantum system, and the other components of the QW system is taken as the large environment. With the measured value of the non-Markovianity for the coin, we can conjecture which kinds of noise emerges in the one and two dimensional QWs.

## Introduction

Quantum walk (QW) has been employed as a useful tool in the study of quantum algorithms and quantum communication^1–17^. For the quantum coherence is introduced into the coin operator in the QW, the variance of the position distribution in the QW displays the quadratical dependence on time; while in the classical walk (CW) system, the variance of the position distribution increases linearly with time. It means that the walker of the QW system can go further in the position space than the walker of the CW system at the same time^3–5^. Such properties have been applied to design the quantum algorithms with the QW^9^. The quantum search algorithms based on the QW display the speedup over the classical algorithms. It only requires ${\mathscr{O}}(\sqrt{N})$ and ${\mathscr{O}}(\sqrt{N\,\mathrm{ln}\,N})$ steps to find a desired item out of *N* items on the hypercube and two-dimensional lattice, respectively^6–17^. However, due to the inevitable interaction from the environment, the decoherence will emerge in the QW system^18–38^. Because of the loss of the quantum coherence in the QW system, the dynamic properties of the QW system will exhibit the similar behaviors as that of the CW system. The variance of the position distribution in the QW shows the linear time dependence when the strong decoherence is introduced into the walk, and the critical decoherence strength for the change from QW to CW depends on the type of noise^19, 30, 38^. Meanwhile, the advantage from the quantum coherence in the quantum search algorithms disappears when the decoherence emerges in the walk^37^. Up to now, considering the decoherence, there has not been an efficient quantum algorithms based on the QW.

Although the decoherence is detrimental to many functions of the QW, in some respects, the decoherence can show many benefits. With the very weak decoherence in the walk, the uniform distribution in the position of the walk is achieved and the desirable quantum speedup is enhanced^21^. The efficiency of quantum transport can be improved with the aid of decoherence^36^. The advantage of decoherence only emerges in the QW with certain noises. Besides, due to impacts from different noises, the different quantum algorithms suiting for different noises are required. In this sense, the sensing of the existence of decoherence and the discrimination among different types of decoherence in the QW system are very important. Such sensing of the QW can help us to develop and choose a feasible quantum algorithm based on the QW for the practical use.

To sense the properties of the QW, it is straightforward to measure the position distribution and the coin populations in the total QW system and get all of the information about it^19, 30, 38^. However, due to the extended infinite space of the position, it is hard for us to perform such measurement on the position space and get the knowledge of the total walk. So it will be very useful if we can develop the quantum sensing method to uncover the properties of the QW with accessing only small part of the QW system. So far the way to distinguish different noises in the walk has been discussed rarely. On the other hand, in optical systems, when taking the optical polarization degrees of freedom and the optical momentum degrees of freedom as the open quantum system and the environment respectively, some researchers have interpreted that by only measuring the non-Markovianity of the polarization degrees of freedom, the existence of the initial correlation between the system and environment can be revealed^39–50^.

Motivated by the investigations above, in this work we introduce the non-Markovianity of a small quantum system to sense the inaccessible total QW system. In our study, we take the two-level coin in the QW system as the open quantum system. By examining the populations of the coin, we can conjecture whether the decoherence emerges in the QW system. Furthermore, in our discussion, we take the broken line noise and coin-decoherence as two different kinds of decoherence. These two kinds of decoherence have been widely used in the description of the dynamics of the QW containing noises^18–38^. We find that, when these two different noises affect the one or two dimensional QW, the non-Markovianity of the coin will exhibit different time evolution behaviors with the system-environment coupling strength. It means that we can infer these two different types of noises in the QW by detecting the non-Markovianity of the coin only and without measuring the total QW system. Though the non-Markovian dynamics in the QW has been mentioned already^51–53^, the studies there mainly focused on the evolution dynamics of the coin, and had no discussion about the discrimination of different noises in the QW.

The organization of our work is as follows: the Sec. *Results* contains two subsections, in Subsec. *The populations of the coin in one and two dimensional QWs with or without noises*, we present the populations of the coin in the one and two dimensional QWs with or without noises, two different kinds of noises are discussed in this subsection. In Subsec. *Quantum sensing of different noises in the one and two dimensional QWs*, we study the non-Markovianity of the coin with different decoherence strengths. Our results reveal that by detecting the non-Markovianity of the coin, we can conjecture which kinds of noise exists in the system. The calculation details associated with the derivation of the populations of coin have been addressed in the Sec. *Methods*. Later, we provide our conclusion and discuss the future application of our findings in Sec. *Discussion and Conclusion*.

## Results

The total system of QW contains the coin and the position. In our discussion, the Hilbert space for the coin is spanned by |*L*〉 and |*R*〉, and the coin operator *C* is

1 $$C=(\begin{array}{cc}\cos \,\theta  & \sin \,\theta \\ \sin \,\theta  & -\cos \,\theta \end{array}).$$

Here, the parameter *θ* in the coin operator *C* is the rotation angle of the coin, and $\theta \in [0,2\pi ]$. For one dimensional QW, we assume that the Hilbert space for the position space is spanned by basis along *x*-direction |*x*〉, $x\in {\mathscr{Z}}$. And for two dimensional QW, the Hilbert space for the position space is spanned by basis along two orthogonal directions (*x*-direction |*x*〉, $x\in {\mathscr{Z}}$ and *y*-direction |*y*〉, $y\in {\mathscr{Z}}$). In one dimensional QW, the conditional shift operator ${S}_{x}^{^{\prime} }$ without noises is

2 $${S}_{x}^{^{\prime} }=\sum _{x=-\infty }^{\infty }\,|x+1,R\rangle \langle x,R|+|x-1,L\rangle \langle x,L|.$$

For the description of conditional shift operators in the two dimensional QW, the shift operator *S*_*y*_ is needed besides *S*_*x*_. The two conditional shift operators *S*_*x*_ and *S*_*y*_ without noises are

3a $${S}_{x}=\sum _{x,y=-\infty }^{\infty }\,|x+1,y,R\rangle \langle x,y,R|+|x-1,y,L\rangle \langle x,y,L|,$$

3b $${S}_{y}=\sum _{x,y=-\infty }^{\infty }\,|x,y+1,R\rangle \langle x,y,R|+|x,y-1,L\rangle \langle x,y,L|.$$

### The populations of the coin in one and two dimensional QWs with or without noises

In this subsection, we study the populations of the coin in the QWs without noises firstly. Then the two different kinds of noises are introduced into the walk, and we present the populations of the coin in the QWs with noises. As reported in ref. 51, we can find that in one dimensional QW without noises, the populations of the coin in the infinite time limit depend explicitly on the initial state of the coin. In the following, we study the populations of the two-level coin in the two dimensional QW without noises.

The state of total two dimensional QW system comprising the coin and position is addressed as

4 $$\begin{array}{ll}|{\rm{\Psi }}(t)\rangle  & =\sum _{x=-\infty }^{\infty }\,\sum _{y=-\infty }^{\infty }\,{a}_{x,y}(t)|x\rangle |y\rangle |R\rangle +{b}_{x,y}(t)|x\rangle |y\rangle |L\rangle .\end{array}$$

For one step evolution of the two dimensional QW, we assume that we apply the coin operator *C* firstly to the coin space, followed by the conditional shift operator along *x*-direction *S*_*x*_; then the coin operator *C* is applied on the coin space again, followed by the conditional shift operator along *y*-direction *S*_*y*_. The unitary operator for one step evolution in the two dimensional QW is $U={S}_{y}(I\otimes C){S}_{x}(I\otimes C)$. Such QW has been demonstrated to recover the phenomenon of the traditional two dimensional QW with a four-level coin^38, 54–56^. Due to the small space of the coin, such QW has a large advantage in designing the quantum algorithms^54, 56^.

After one step evolution, the coefficients *a*_*x*,*y*_(*t*) and *b*_*x*,*y*_(*t*) of the state $|{\rm{\Psi }}(t)\rangle$ at time *t* + 1 change as

5a $$\begin{array}{rcl}{a}_{x,y}(t+1) & = & {a}_{x-1,y-1}(t)\,{\cos }^{2}\,\theta +{a}_{x+\mathrm{1,}y-1}(t)\,{\sin }^{2}\,\theta \\  &  & +{b}_{x-\mathrm{1,}y-1}(t)\,\sin \,\theta \,\cos \,\theta -{b}_{x+\mathrm{1,}y-1}(t)\,\cos \,\theta \,\sin \,\theta ,\end{array}$$

5b $$\begin{array}{rcl}{b}_{x,y}(t+\mathrm{1)} & = & {a}_{x-\mathrm{1,}y+1}(t)\,\cos \,\theta \,\sin \,\theta -{a}_{x+\mathrm{1,}y+1}(t)\,\sin \,\theta \,\cos \,\theta \\  &  & +{b}_{x-\mathrm{1,}y+1}(t)\,{\sin }^{2}\,\theta +{b}_{x+\mathrm{1,}y+1}(t)\,{\cos }^{2}\,\theta .\end{array}$$

The population for the state |*R*〉 of the coin is ${P}_{R}(t)=\sum _{x,y=-\infty }^{\infty }\,|{a}_{x,y}(t){|}^{2}$, and the population for the state |*L*〉 of the coin is ${P}_{L}(t)=\sum _{x,y=-\infty }^{\infty }\,|{b}_{x,y}(t){|}^{2}$, the time evolutions for *P*_*R*_(*t*) and *P*_*L*_(*t*) are

6 $$[\begin{array}{c}{P}_{R}(t+1)\\ {P}_{L}(t+\mathrm{1)}\end{array}]=(\begin{array}{cc}{\cos }^{4}\,\theta +{\sin }^{4}\,\theta  & 2\,{\cos }^{2}\,\theta \,{\sin }^{2}\,\theta \\ 2\,{\cos }^{2}\,\theta \,{\sin }^{2}\,\theta  & {\sin }^{4}\,\theta +{\cos }^{4}\,\theta \end{array})\,[\begin{array}{c}{P}_{R}(t)\\ {P}_{L}(t)\end{array}]+A(t)(\begin{array}{c}1\\ -1\end{array}),$$

with

7 $$\begin{array}{rcl}A(t) & = & 2{\rm{Re}}[{Q}_{1}(t)]\,{\cos }^{2}\,\theta \,{\sin }^{2}\,\theta -2{\rm{Re}}[{Q}_{2}(t)]\,{\cos }^{3}\,\theta \,\sin \,\theta \\  &  & +2{\rm{Re}}[{Q}_{3}(t)]\,{\sin }^{3}\,\theta \,\cos \,\theta -2{\rm{Re}}[{Q}_{4}(t)]\,{\sin }^{2}\,\theta \,{\cos }^{2}\,\theta \\  &  & +2{\rm{Re}}[{Q}_{5}(t)]\,\cos \,\theta \,\sin \,\theta ({\cos }^{2}\,\theta -{\sin }^{2}\,\theta ).\end{array}$$

Here, ${Q}_{i}(i=1\ldots \mathrm{5)}$ is expressed as ${Q}_{1}(t)={\sum }_{x,y=-\infty }^{\infty }\,{a}_{x+\mathrm{1,}y-1}(t){a}_{x-\mathrm{1,}y-1}^{\ast }(t),$ ${Q}_{2}(t)={\sum }_{x,y=-\infty }^{\infty }{a}_{x-\mathrm{1,}y-1}(t)$ ${b}_{x+\mathrm{1,}y-1}^{\ast }(t),$ ${Q}_{3}(t)={\sum }_{x,y=-\infty }^{\infty }\,{a}_{x+\mathrm{1,}y-1}(t){b}_{x-\mathrm{1,}y-1}^{\ast }(t),$ ${Q}_{4}(t)={\sum }_{x,y=-\infty }^{\infty }\,{b}_{x+\mathrm{1,}y-1}(t){b}_{x-\mathrm{1,}y-1}^{\ast }(t),$ ${Q}_{5}(t)={\sum }_{x,y=-\infty }^{\infty }$ ${a}_{x-\mathrm{1,}y-1}(t){b}_{x-\mathrm{1,}y-1}^{\ast }(t)\,$=${\sum }_{x,y=-\infty }^{\infty }\,{a}_{x+\mathrm{1,}y-1}(t){b}_{x+\mathrm{1,}y-1}^{\ast }(t)$. Considering the obtained expressions *P*_*R*_(*t*) and *P*_*L*_(*t*) for the coin in Eq. 6, when time *t* approaches infinity, we find that the populations of the coin *P*_*R*_(*t*) and *P*_*L*_(*t*) are affected by the interference term *A*(*t*→∞),

8a $${{\rm{\Pi }}}_{R}={P}_{R}(t\to \infty )=\frac{1}{2}+\frac{A(t\to \infty )}{4\,{\cos }^{2}\,\theta \,{\sin }^{2}\,\theta },$$

8b $${{\rm{\Pi }}}_{L}={P}_{L}(t\to \infty )=\frac{1}{2}-\frac{A(t\to \infty )}{4\,{\cos }^{2}\,\theta \,{\sin }^{2}\,\theta }.$$

We can find that for one dimensional QW, the contribution to the populations of the coin (*P*_*L*_(*t*) and *P*_*R*_(*t*)) is related to the interference term which is associated with the coefficients *a*_*x*_ and *b*_*x*_^51^. From the interference terms ${Q}_{i}(i=1\ldots \mathrm{5)}$ in Eq. 7, it is obvious that the interference is not only from the coefficients *a*_*x*,*y*_ and *b*_*x*,*y*_, but also from the coefficients *a*_*x*+1,*y*_ and *a*_*x*−1,*y*_ (*b*_*x*+1,*y*_ and *b*_*x*−1,*y*_). Furthermore, it is clearly seen that for 2 by 2 matrix in Eq. 6, the elements are all positive and the sum over any column or row of this matrix is equal to 1. The transition between the states |*R*〉 and |*L*〉 from such 2 by 2 matrix can be seen as a Markovian process. While in Eq. 6, another term associated with *A*(*t*) is added. In this case, the time evolution for the populations of the coin (Eq. 6) cannot be interpreted as a simple Markovian process.

Moreover, by applying Fourier transform, we will derive the populations of the coin in the two dimensional QW when time approaches infinity. We will compare the results from the Fourier transform method with the results aforementioned (Eq. 8a and 8b). In our discussion below, we take the parameter *θ* of the coin operator *C* as *θ* = *π*/4 (that is the Hadamard matrix). After taking the Fourier transform in the position spaces |*x*〉 and |*y*〉, we can obtain

9a $$|x\rangle ={\int }_{-\pi }^{\pi }\,\frac{dk}{2\pi }{e}^{-ikx}|k\rangle ,$$

9b $$|y\rangle ={\int }_{-\pi }^{\pi }\,\frac{dp}{2\pi }{e}^{-ipy}|p\rangle .$$

By taking such expressions, the shift along *x* and *y*-directions can be interpreted as ref. 38

10 $$\sum _{x,y}\,|x+{l}_{1},y+{l}_{2}\rangle \langle x,y|=\frac{1}{{\mathrm{(2}\pi )}^{2}}{\int }_{-\pi }^{\pi }\,{\int }_{-\pi }^{\pi }\,dkdp{e}^{-i{l}_{1}k-i{l}_{2}p}|k,p\rangle \langle k,p|.$$

**Figure 1:**
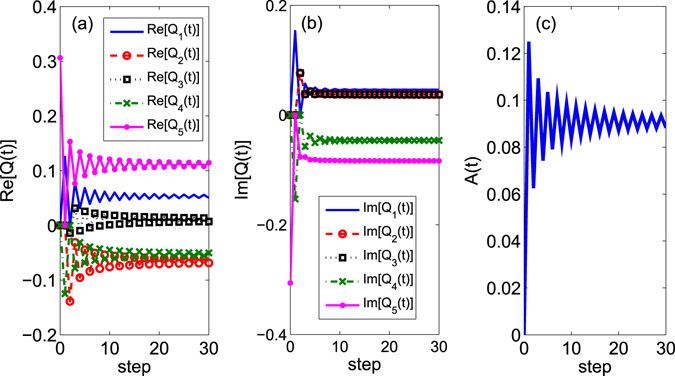
The step evolution of ${Q}_{i}(t)\,(i=1\ldots \mathrm{5)}$ and *A*(*t*) in Eq. 7. The initial total state for this two dimensional QW is taken as $[\cos \,\frac{\pi }{6}|R\rangle +\exp (i\frac{\pi }{4})\,\sin \,\frac{\pi }{6}|L\rangle ]\,{|0\rangle }_{x}{|0\rangle }_{y}$. (**a**) The step evolution of the real part of *Q*_*i*_(*t*); (**b**) The step evolution of the imaginary part of *Q*_*i*_(*t*); (**c**) The step evolution of *A*(*t*).

The initial state for the coin takes the form as $|\varphi \mathrm{(0)}\rangle =\,\cos \,\frac{\gamma }{2}|R\rangle +{e}^{i\delta }\,\sin \,\frac{\gamma }{2}|L\rangle$. The coin state at time *t* approaching infinity is addressed as

11 $${\rho }_{c}(t\to \infty )=(\begin{array}{cc}{{\rm{\Pi }}}_{R}^{^{\prime} } & {Q}_{0}\\ {Q}_{0}^{\ast } & {{\rm{\Pi }}}_{L}^{^{\prime} }\end{array}),$$

with ${{\rm{\Pi }}}_{R}^{^{\prime} }=\frac{1}{2}+\frac{0.36338}{2}\,\cos \,\gamma$, ${{\rm{\Pi }}}_{L}^{^{\prime} }=\frac{1}{2}-\frac{0.36338}{2}\,\cos \,\gamma$ and ${Q}_{0}=\frac{0.36338}{2}\,\cos \,\delta \,\sin \,\gamma -\frac{i}{2}0.27324\,\sin \,\delta \,\sin \,\gamma$. Here, ${{\rm{\Pi }}}_{R}^{^{\prime} }=$ $\mathop{\mathrm{lim}}\limits_{t\to \infty }{P}_{R}(t)=\mathop{\mathrm{lim}}\limits_{t\to \infty }\,{{\sum }_{x,y=-\infty }^{\infty }|{a}_{x,y}(t)|}^{2},$ ${{\rm{\Pi }}}_{L}^{^{\prime} }=\mathop{\mathrm{lim}}\limits_{t\to \infty }\,{P}_{L}(t)=\mathop{\mathrm{lim}}\limits_{t\to \infty }\,{\sum }_{x,y=-\infty }^{\infty }{|{b}_{x,y}(t)|}^{2},$ and ${Q}_{0}=\mathop{\mathrm{lim}}\limits_{t\to \infty }\,{\sum }_{x,y=-\infty }^{\infty }\,{a}_{x,y}$ $(t){b}_{x,y}^{\ast }(t)$. The detailed derivation of equations above can be found in Sec. *Methods*. In Fig. 1, we provide the example to show that when the time becomes large, our analytic results about the populations of the coin (Eq. 8a and 8b) coincide with the numerical results obtained from the Fourier transform (Eq. 11). We choose the parameter *θ* = *π*/4, that means the coin is the Hadamard matrix in the derivation of Eqs 6, 8a and 8b. The initial coin state is $|\varphi \mathrm{(0)}\rangle =\,\cos \,\frac{\pi }{6}|R\rangle +\exp \,(i\frac{\pi }{4})\,\sin \,\frac{\pi }{6}|L\rangle$. Moreover, it clearly shows that when the time *t* becomes large, the term *Q*_5_(*t*) (the magenta solid dotted line in Fig. 1(a,b)) is close to the term *Q*_0_ from the Fourier transform method. The real part and imaginary part of *Q*_5_(*t*) at *t* = 30 are 0.1145 and −0.08361, respectively. When substituting *γ* = *π*/3 and *δ* = *π*/4 into *Q*_0_, we obtain *Q*_0_ = 0.1113 − *i* · 0.0837. These two values ${Q}_{5}(t=\mathrm{30)}$ and *Q*_0_ are nearly the same. Besides the value of *Q*_5_(*t*) is close to *Q*_0_, when the time approaches infinity, the time evolution of *A*(*t*) is addressed in Fig. 1(c). In our figure, the term *A*(*t*) at time *t* = 30 is equal to 0.08826, then the populations of the coin *P*_*R*_ and *P*_*L*_ from Eqs 6, 8a and 8b are 0.5883 and 0.4117, respectively. When we put *γ* = *π*/3 and *δ* = *π*/4 into Eq. 11, the values for ${{\rm{\Pi }}}_{R}^{^{\prime} }$ and ${{\rm{\Pi }}}_{L}^{^{\prime} }$ from the Fourier transform method are 0.5908 and 0.4092, respectively. It means that our derivation based on the step evolution equation (Eqs 6, 8a and 8a) coincides with that method related to the Fourier transform (Eq. 11).

Next, we will study the populations of the coin when the total QW system is affected by the noises. The populations of the coin with the noises will be compared to that without the noises. Here, two different types of noises are discussed. One is the broken line noise, which describes a nonseparable noise in the coin-position system; the other is the coin-decoherence, which only emerges in the coin space and is seen as a separable noise. These two types of noises are common noises in the QW and have been widely discussed^18–38^. For one dimensional QW with the broken line noise, the populations of the coin when time approaches infinity have been presented as ${\rho }_{c}(t\to \infty )=\mathrm{1/2}(|L\rangle \langle L|+|R\rangle \langle R|)$ ^51^. When the coin-decoherence is introduced into the one dimensional QW, we present the reduced density matrix of the coin *ρ*_*c*_(*t*) by using the Fourier transform method in Sec. *Methods*. When the time approaches infinity, ${\rho }_{c}(t\to \infty )=\mathrm{1/2}(|L\rangle \langle L|+|R\rangle \langle R|)$.

Moreover, we study the populations of the coin in the two dimensional QWs with noises. Firstly, the broken line noise is introduced into the walk. We assume that the broken line noise appears only in the *x*-direction, and four possible evolutions of the two dimensional QW involving decoherence are included^38^. The explicit form of evolution operator ${ {\mathcal L} }_{k,p}$ can be found in Sec. *Methods*. From the calculation in Sec. *Methods*, we find that no matter what the initial coin state is, the density matrix for the coin *ρ*_*c*_(*t*) in the infinite time limit is

12 $${\rho }_{c}(t\to \infty )=\frac{1}{2}(\begin{array}{cc}1 & 0\\ 0 & 1\end{array}).$$

Next, we study the populations of the coin in the two dimensional QW with the introduction of the coin-decoherence. In our discussion, we assume that coin-decoherence emerges with the probability *f* before each step of the walk, then the walker moves along the *x*-direction and *y*-direction in sequence. The operator ${ {\mathcal L} }_{k,p}$ describing one step evolution of the two dimensional QW with the coin-decoherence has been presented in Sec. *Methods*. Based on the calculation in Sec. *Methods*, it is clear that no matter what the initial coin state is, when the time approaches infinity, the reduced density matrix for the coin *ρ*_*c*_(*t*) is

13 $${\rho }_{c}(t\to \infty )=\frac{1}{2}(\begin{array}{cc}1 & 0\\ 0 & 1\end{array}).$$

Based on the discussion above, we have considered two different kinds of noises in the one or two dimensional QWs. One is the broken line noise and the other is the coin-decoherence. Our calculation results have revealed that when time *t* lasts long enough, the state of coin will approach $\frac{1}{2}(|R\rangle \langle R|+|L\rangle \langle L|)$, no matter what the initial coin state is. While, if we do not consider the effect of the noise, when time approaches infinity, the populations of the coin in one or two dimensional QWs depend on the initial coin state $|\varphi \mathrm{(0)}\rangle$ explicitly (Eqs 8a, 8b and 11). It means that for one or two dimensional QWs, from our obtained populations of the coin, we can conjecture whether the total QW system is affected by these two common noises.

### Quantum sensing of different noises in the one and two dimensional QWs

As mentioned above, by only detecting the populations of the coin, we can sense the existence of the two common noises in the QW. If there is no noise in the walk, the populations of the coin depend explicitly on the initial state of the coin. In contrast, when there exists the broken line noise or the coin-decoherence in the walk, the coin approaches the state $\frac{1}{2}(|R\rangle \langle R|+|L\rangle \langle L|)$ with the step evolution. Considering the importance of discrimination of these two types of noises in designing the quantum algorithms^9–14, 17, 37, 38^, we will propose to sense the noise in the QW by taking the coin as the open system and only measuring the non-Markovianity of the coin (the density matrix of coin only has 2 by 2 elements).

In our proposal, the non-Markovianity of the coin in the QW is detected. Due to the interaction between the coin and the position in each step evolution of the walk, the information obtained from the coin can reveal the properties of the whole quantum walk system. We find that when the one or two dimensional QW system is affected by different types of noises, the non-Markovianity of the coin exhibits different behaviors. In the discussion below, two common noises are taken into the walk, one is the broken line noise and the other is coin-decoherence. When the evolution of the QW system incorporates one of such two types of noises, we can conjecture that which noise is contained in the walk by only detecting the non-Markovianity of the coin.

The non-Markovianity ${\mathscr{N}}(t\to \infty )$ for quantum processes in open system is defined as refs 40 and 41

14 $${\mathscr{N}}(t\to \infty )=\mathop{{\rm{\max }}}\limits_{{\rho }_{1},{\rho }_{2}}{\int }_{\sigma  > \mathrm{0,}\tau \in [0,t]}\,\sigma (\tau ,{\rho }_{\mathrm{1,2}}\mathrm{(0))}d\tau ,$$

where the rate of change of the trace distance $\sigma (t,{\rho }_{\mathrm{1,2}}\mathrm{(0))}$ is addressed as

15 $$\sigma (t,{\rho }_{\mathrm{1,2}}\mathrm{(0))}=\frac{d}{dt}D[{\rho }_{1}(t)-{\rho }_{2}(t)].$$

The expression $D[{\rho }_{1}(t)-{\rho }_{2}(t)]$ denotes the trace distance between the two density matrices *ρ*_1_(*t*) and *ρ*_2_(*t*). In Eq. 14, the integral is taken when the trace distance between *ρ*_1_(*t*) and *ρ*_2_(*t*) increases with the time, and the maximization is performed over all of possible initial states *ρ*_1_(0) and *ρ*_2_(0). Due to our calculation power, we cannot obtain the asymptotic value of ${\mathscr{N}}(t)$ with *t*→∞ to evaluate the non-Markovian process precisely. Our results below associated with ${\mathscr{N}}(t)$ in the finite time can clearly reveal the different non-Markovian behaviors in the walk with different kinds of noises. Based on the different features of the non-Markovianity ${\mathscr{N}}(t)$, we can easily judge what kinds of noise emerges in the walk.

#### The sensing in the one dimensional QW

**Figure 2:**
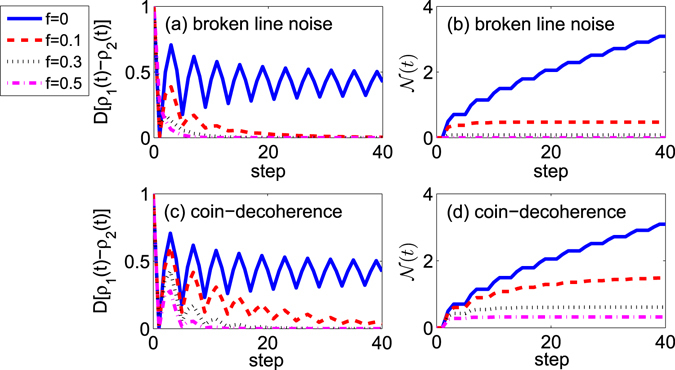
The one dimensional QW with different strengths of decoherence. Blue solid, *f* = 0; Red dashed, *f* = 0.1; Black dotted, *f* = 0.3; Magenta dotted dashed, *f* = 0.5. (**a**,**b**) The broken line noise is considered in the walk. (**a**) The step evolution of the trace distance between the coin states *ρ*_1_(*t*) and *ρ*_2_(*t*). The initial coin states are chosen as ${\rho }_{1}\mathrm{(0)}=|R\rangle \langle R|$ and ${\rho }_{2}\mathrm{(0)}=|L\rangle \langle L|$. (**b**) The non-Markovianity ${\mathscr{N}}(t)$ of the coin with the step evolution in the walk. (**c**,**d**) The coin-decoherence is introduced into the walk. (**c**) The trace distance between the coin states *ρ*_1_(*t*) and *ρ*_2_(*t*). The initial coin states are chosen as ${\rho }_{1}\mathrm{(0)}=|R\rangle \langle R|$ and ${\rho }_{2}\mathrm{(0)}=|L\rangle \langle L|$. (**d**) The non-Markovianity of the coin with the step evolution in the walk.

For one dimensional QW, as presented before, we have found that when the noise is not introduced, the dynamics of the populations for the coin shows the explicit dependence on the initial coin state. In comparison, when any one noise is taken into account, the state for coin reaches the asymptotic state $\frac{1}{2}|R\rangle \langle R|+\frac{1}{2}|L\rangle \langle L|$ in the infinite time whatever the initial coin state is. Next, the non-Markovianity ${\mathscr{N}}(t)$ and the trace distance $D[{\rho }_{1}(t)-{\rho }_{2}(t)]$ for one dimensional QWs are presented in Fig. 2. We assume that the walker starts at the original point. The simulation has been done with all of possible initial states for the coin, and we find that the maximum value in the integral Eq. 14 is obtained with the initial states for the coin being |*R*〉 and |*L*〉, respectively. So in our simulation, the initial states of the coin for the evaluation of trace distance *D* are taken as ${\rho }_{1}\mathrm{(0)}=|R\rangle \langle R|$ and ${\rho }_{2}\mathrm{(0)}=|L\rangle \langle L|$.

In Fig. 2, the trace distance $D[{\rho }_{1}(t)-{\rho }_{2}(t)]$ and the non-Markovianity with different strengths of decoherence are addressed. Two different kinds of noises are provided. In Fig. 2(a,b), the broken line noise is introduced into the walk; in Fig. 2(c,d), the coin-decoherence emerges in the one dimensional QW. As addressed in Fig. 2(b,d), for the one dimensional QW, with the strength of decoherence becoming larger, the non-Markovianity ${\mathscr{N}}(t)$ of the open system (coin) becomes smaller. When the strength of the broken line noise is 0.3 (see Fig. 2(b)), the non-Markovianity of the coin decreases to a very small value. When the strength of decoherence increases to *f* = 0.5, the dynamics of the coin in the one dimensional QW involving the broken line noise changes to a Markovian process. When the coin-decoherence is introduced into the walk (see Fig. 2(d)), we can find that, with the strength of decoherence becoming larger, the coin displays less non-Markovian behavior. While, compared with the one dimensional walk with broken line noise, even though the strength of decoherence increases to *f* = 0.5, the coin in the one dimensional QW with coin-decoherence still undergoes a non-Markovian process. By detecting the non-Markovianity ${\mathscr{N}}(t)$ of the coin, we can conjecture that whether the broken line noise or the coin-decoherence emerges in the one dimensional QW.

**Figure 3:**
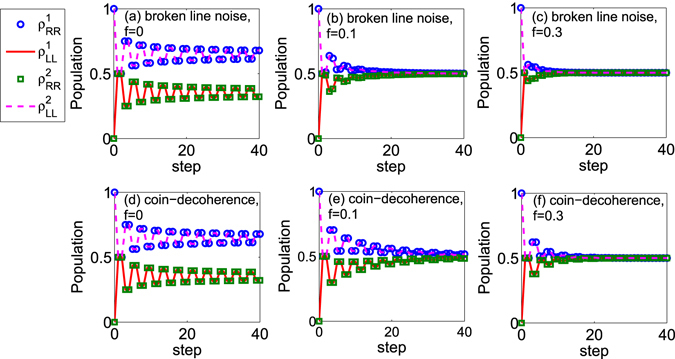
The populations of the coin in the one dimensional QW with different decoherence strengths. Blue circles and red solid line describe the populations of the coin at the state |*R*〉 and |*L*〉, respectively; the initial coin state is ${\rho }_{1}\mathrm{(0)}=|R\rangle \langle R|$. Green rectangles and magenta dashed line represent the populations of the coin at the state |*R*〉 and |*L*〉, respectively; the initial coin state is ${\rho }_{2}\mathrm{(0)}=|L\rangle \langle L|$. (**a**–**c**) The broken line noise is considered in the walk. (**d**–**f**) The coin-decoherence emerges in the walk.

Later, we analyze the populations of the coin when the decoherence is considered. In Fig. 3, we provide the populations of the coin with the step evolution in the walk. Two different kinds of noises are introduced. In Fig. 3(a–c), the broken line noise emerges in the walk. For Fig. 3(d–f), the coin-decoherence appears in the walk. Different strengths of noise are addressed. When the strength of decoherence satisfies *f* = 0 (see Fig. 3(a,d)), which means there is no noise in the walk, the populations of coin at the state |*R*〉 and |*L*〉 are not same. While, when any one noise emerges in the walk (see Fig. 3(b,c,e,f)), with the increase of the step, the populations of the coin at the state |*R*〉 and |*L*〉 are nearly equal. Such results have been obtained from our aforementioned discussion. Furthermore, considering the two initial coin states here (|*R*〉 and |*L*〉) are same as that used in the description of the non-Markovianity ${\mathscr{N}}(t)$ (Fig. 2), we find that when the strength of decoherence increases, the amplitudes of oscillations for the populations of the coin reduce. For the oscillations of the populations are directly related to the increase of trace distance $D[{\rho }_{1}(t)-{\rho }_{2}(t)]$, it means that the smaller value of non-Markovianity ${\mathscr{N}}(t)$ can be found with a larger strength of decoherence, which coincides with the results in Fig. 2. When the noise is introduced into the walk, at the same strength of decoherence, the oscillations of the populations of the coin in the QW with the broken line noise are weaker than that in the QW with the coin-decoherence (from Fig. 3(b,e), *f* = 0.1; Fig. 3(c,f), *f* = 0.3). These oscillation behaviors agree with the amplitudes of non-Markovianity of the coin ${\mathscr{N}}(t)$ in one dimensional QWs with different kinds of noises (see Fig. 2(b,d)).

#### The sensing in the two dimensional QW

**Figure 4:**
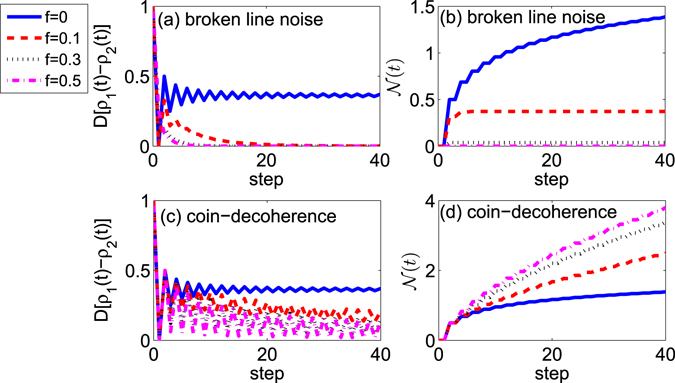
The two dimensional QW with different strengths of decoherence. Blue solid, *f* = 0; Red dashed, *f* = 0.1; Black dotted, *f* = 0.3; Magenta dotted dashed, *f* = 0.5. (**a**,**b**) The broken line noise is considered in the walk. (**a**) The trace distance between the coin states *ρ*_1_(*t*) and *ρ*_2_(*t*). The initial coin states are chosen as ${\rho }_{1}\mathrm{(0)}=|R\rangle \langle R|$ and ${\rho }_{2}\mathrm{(0)}=|L\rangle \langle L|$. (**b**) The non-Markovianity of the coin with the step evolution in the walk. (**c**,**d**) The coin-decoherence appears in the walk. (**c**) The trace distance between the coin states *ρ*_1_(*t*) and *ρ*_2_(*t*). The initial coin states are chosen as ${\rho }_{1}\mathrm{(0)}=|R\rangle \langle R|$ and ${\rho }_{2}\mathrm{(0)}=|L\rangle \langle L|$. (**d**) The non-Markovianity of the coin with the step evolution in the walk.

In what follows, we study the non-Markovianity of the coin in the two dimensional QW. The populations of the coin in the two dimensional QWs with or without noises have been discussed above. When the broken line noise or coin-decoherence emerges in the walk, the populations of coin at the state |*R*〉 and |*L*〉 are close to each other with the step evolution of the walk. In Fig. 4, we present the non-Markovianity of the coin ${\mathscr{N}}(t)$ in the walk with two different kinds of noises. We have numerically demonstrated that the maximum value for the integral Eq. 14 in the two dimensional QWs can be reached with the two initial coin states chosen as |*R*〉 and |*L*〉. In comparison with the non-Markovianity for the coin in the one dimensional QWs, the different behaviors of the non-Markovianity for the coin emerge in the two dimensional QWs with the decoherence. As shown in Fig. 4(b,d), for the two dimensional QWs, when the broken line noise appears in the walk, the value of ${\mathscr{N}}(t)$ decreases with the increase of the strength of decoherence, while, when there is the coin-decoherence in the walk, the value of ${\mathscr{N}}(t)$ increases with the increase of the strength of decoherence.

**Figure 5:**
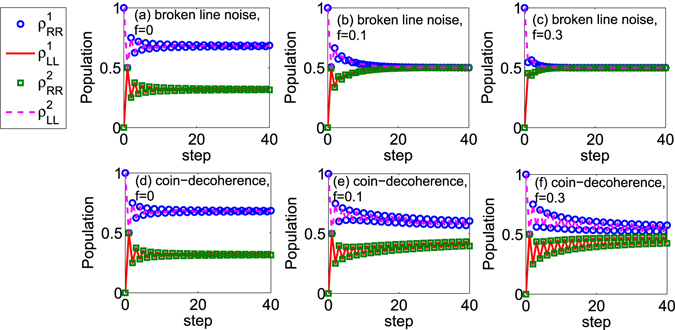
The populations of the coin in the two dimensional QW with different decoherence strengths. Blue circles and red solid lines stand for the populations of the coin at the state |*R*〉 and |*L*〉, respectively; the initial state of the coin is ${\rho }_{1}\mathrm{(0)}=|R\rangle \langle R|$. Green rectangles and magenta dashed line depict the populations of the coin at the states |*R*〉 and |*L*〉, respectively; the initial state of the coin is ${\rho }_{2}\mathrm{(0)}=|L\rangle \langle L|$. (**a**–**c**) The broken line noise is considered in the walk. (**d**–**f**) The coin-decoherence emerges in the walk.

To illustrate the different behaviors of the non-Markovianity with the decoherence, we provide the populations of the coin with step evolution in the two dimensional QWs, see Fig. 5. From Fig. 5(b,c,e,f), we find that when there exists one noise in the two dimensional QWs, the populations for the coin at the state |*R*〉 and |*L*〉 approach the same value ${\rho }_{RR}^{k=\mathrm{1,2}}={\rho }_{LL}^{k=\mathrm{1,2}}=0.5$. These numerical results are consistent with the results obtained above. For the broken line noise is introduced into the walk (Fig. 5(b,c)), the amplitudes of the oscillations in the populations of the coin decrease with the increase of the decoherence strength. Such attenuation in the oscillations of the populations of the coin reduces the increase of the trace distance between the state *ρ*_1_(*t*) and *ρ*_2_(*t*), and leads to the decrease of the non-Markovianity ${\mathscr{N}}(t)$ for the coin. While, when the coin-decoherence emerges in the two dimensional QW, we find that with the increase of the decoherence strength, the amplitudes of the oscillations in the populations of the coin increase. Such increases of oscillations lead to the increase of the value of the non-Markovianity.

Based on the statements above, we can find that by detecting the populations of the coin, we can judge whether there exists the decoherence in the QW. In our study, the two widely discussed noises have been introduced. One is the broken line noise, which is the nonseparable noise in the coin and position space; the other is the coin-decoherence, which appears only in the coin space. For there exists one of these two common noises in the walk, the state of the coin reaches $\frac{1}{2}|R\rangle \langle R|+\frac{1}{2}|L\rangle \langle L|$ when the step of the walk becomes large. Then, when one of two common noises appears in the walk, by examining the value of non-Markovianity of the coin ${\mathscr{N}}(t)$ in the walk, we can conjecture what kinds of noise exists in the walk. For one dimensional QW, when the strength of decoherence increases, the value ${\mathscr{N}}(t)$ of the coin shows a faster decrease in the QW with the broken line noise than that in the QW with the coin-decoherence. If the decoherence becomes strong enough (*f* > 0.3), the quantum dynamics of the coin in the QW with the broken line noise changes to a Markovian process; while, the non-Markovianity ${\mathscr{N}}(t)$ of the coin in the QW with the coin-decoherence still exhibits a non-zero value when the decoherence increases. For two dimensional QW with the broken line noise or coin-decoherence, the behaviors of the non-Markovianity for the coin display different features. The value of non-Markovianity for the coin decreases when the broken line noise is introduced; while, when the coin-decoherence emerges in the walk, the value of non-Markovianity for the coin is higher than that without noises. So by detecting the value of non-Markovianity ${\mathscr{N}}(t)$ for the coin, and comparing the obtained ${\mathscr{N}}(t)$ with that for the coin without noises, we can judge which kinds of noise appears in the one and two dimensional QWs.

## Discussion and Conclusion

In this paper, we study how to sense the noise in the total QW system by just detecting the coin in the walk. For the representation of the coin needs only 2 by 2 matrix, and the infinite dimensional space is required to describe the position in the QW system. It is of great value to reveal the features of the total QW system with the observation of the coin only. In our study, two common widely used noises in the QW are presented. By examining the populations of the coin, we can conjecture whether there exists the decoherence in the one or two dimensional QWs. Then we discuss the non-Markovianity of the coin in the QWs with different strengths of decoherence. Our results indicate that with obtained value of the non-Markovianity of the coin, we can judge whether the broken line noise or the coin-decoherence exists in the one and two dimensional QWs.

Although the destructive measurement on the coin is required in our proposed sensing method, our proposal of sensing the noise in the QWs is practical. The disturbances from the destructive measurements on the coin can be eliminated in some implementations of the QWs^57–61^. In these realizations, the optical polarization of the light is taken as the coin. For the realizations of QWs with the classical light involving optical orbital angular momentum (OAM) modes^57^, from the fraction of beam transmitted through the beam splitter to the detector, we can extract the information of the coin at each step of the walk. For the other realizations of QWs with the position space^59–61^, we can obtain the information of the optical polarization (coin) from the detectors at different positions. Though in these realizations of QWs using the position space, the QWs are disturbed after the detections, we can restart the evolutions of the QWs with different steps and obtain the dynamics of the coin in the walks.

Considering the importance of knowledge about the noise in the QW system, our sensing method provided in this work have potential applications in designing the quantum algorithms based on the QW.

## Methods

### The populations of the coin in two dimensional QWs without noises

The Fourier transform method is applied to derive the populations of the coin. That is $|x\rangle ={\int }_{-\pi }^{\pi }\,\frac{dk}{2\pi }{e}^{-ikx}|k\rangle$, $|y\rangle ={\int }_{-\pi }^{\pi }\,\frac{dp}{2\pi }{e}^{-ipy}|p\rangle$. When taking partial trace over the position space, we get the density matrix for the coin at time *t* as

16 $${\rho }_{c}(t)={{\rm{Tr}}}_{x,y}[\rho (t)]=\sum _{x=-\infty }^{\infty }\,\sum _{y=-\infty }^{\infty }\,\langle x,y|\rho (t)|x,y\rangle =\iint \frac{dk}{2\pi }\frac{dp}{2\pi }{ {\mathcal L} }_{k,p}^{t}|\varphi \mathrm{(0)}\rangle \langle \varphi \mathrm{(0)}|.$$

The initial state for the coin takes the form as $|\varphi \mathrm{(0)}\rangle =\,\cos \,\frac{\gamma }{2}|R\rangle +{e}^{i\delta }\,\sin \,\frac{\gamma }{2}|L\rangle$. We introduce one representation that transform one 2 by 2 matrix to one 4 by 1 column vector, and the initial state $|\varphi \mathrm{(0)}\rangle$ for the coin can be described as

17 $$|\varphi \mathrm{(0)}\rangle \langle \varphi \mathrm{(0)}|={r}_{0}I+{r}_{1}{\sigma }_{x}+{r}_{2}{\sigma }_{y}+{r}_{3}{\sigma }_{z}=(\begin{array}{c}{r}_{0}\\ {r}_{1}\\ {r}_{2}\\ {r}_{3}\end{array})=\tilde{O},$$

where *σ*_*x*_, *σ*_*y*_ and *σ*_*z*_ are the Pauli matrices, and *I* is the 2 by 2 identity matrix. For two dimensional QW, the one step evolution for the walk is $U={S}_{y}(I\otimes C){S}_{x}(I\otimes C)$, the operator ${ {\mathcal L} }_{k,p}$ for this two dimensional QW is

18 $${ {\mathcal L} }_{k,p}=(\begin{array}{cccc}1 & 0 & 0 & 0\\ 0 & \cos \,2p & -\cos \,2k\,\sin \,2p & \sin \,2k\,\sin \,2p\\ 0 & \sin \,2p & \cos \,2k\,\cos \,2p & -\cos \,2p\,\sin \,2k\\ 0 & 0 & \sin \,2k & \cos \,2k\end{array}).$$

Considering the time that we concern goes to infinity, we can obtain the eigenvalues of the operator ${ {\mathcal L} }_{k,p}$ and neglect the oscillatory terms. After doing the integral, we obtain the density matrix of the coin in the infinite time as

19 $$\begin{array}{rcl}{\rho }_{c}(t\to \infty ) & = & {\int }_{-\pi }^{\pi }\,{\int }_{-\pi }^{\pi }\,\frac{dk}{2\pi }\frac{dp}{2\pi }{ {\mathcal L} }_{k,p}^{t\to \infty }|\varphi \mathrm{(0)}\rangle \langle \varphi \mathrm{(0)}|\\  & = & (\begin{array}{cccc}1 & 0 & 0 & 0\\ 0 & 0.36338 & 0 & 0\\ 0 & 0 & 0.27324 & 0\\ 0 & 0 & 0 & 0.36338\end{array})\,(\begin{array}{c}{r}_{0}\\ {r}_{1}\\ {r}_{2}\\ {r}_{3}\end{array}).\end{array}$$

When the initial state of the coin is chosen as $|\varphi \mathrm{(0)}\rangle =\,\cos \,(\gamma \mathrm{/2)}|R\rangle +{e}^{i\delta }\,\sin \,(\gamma \mathrm{/2)}|L\rangle$, the coin state at time *t* approaching infinity is addressed as

20 $${\rho }_{c}(t\to \infty )=(\begin{array}{cc}{{\rm{\Pi }}}_{R}^{^{\prime} } & {Q}_{0}\\ {Q}_{0}^{\ast } & {{\rm{\Pi }}}_{L}^{^{\prime} }\end{array}),$$

with

21 $$\begin{array}{rcl}{{\rm{\Pi }}}_{R}^{^{\prime} } & = & \frac{1}{2}+\frac{0.36338}{2}\,\cos \,\gamma ,\\ {{\rm{\Pi }}}_{L}^{^{\prime} } & = & \frac{1}{2}-\frac{0.36338}{2}\,\cos \,\gamma ,\\ {Q}_{0} & = & \frac{0.36338}{2}\,\cos \,\delta \,\sin \,\gamma -\frac{i}{2}0.27324\,\sin \,\delta \,\sin \,\gamma .\end{array}$$

Here, $${{\rm{\Pi }}}_{R}^{^{\prime} }=\mathop{\mathrm{lim}}\limits_{t\to \infty }\,{P}_{R}(t)=\mathop{\mathrm{lim}}\limits_{t\to \infty }\,{\sum }_{x,y=-\infty }^{\infty }|{a}_{x,y}(t){|}^{2}$$, $${{\rm{\Pi }}}_{L}^{^{\prime} }=\mathop{\mathrm{lim}}\limits_{t\to \infty }\,{P}_{L}(t)=\mathop{\mathrm{lim}}\limits_{t\to \infty }\,{\sum }_{x,y=-\infty }^{\infty }|{b}_{x,y}(t){|}^{2}$$, and $${Q}_{0}=\mathop{\mathrm{lim}}\limits_{t\to \infty }$$ $${\sum }_{x,y=-\infty }^{\infty }\,{a}_{x,y}(t){b}_{x,y}^{\ast }(t)$$.

### The populations of the coin in one dimensional QW with coin-decoherence

When the coin-decoherence is introduced into the one dimensional QW, after one step evolution, the reduced density matrix of the coin *ρ*_*c*_(*t* = 1) can be addressed as

22 $${\rho }_{c}(t=\mathrm{1)}={{\rm{Tr}}}_{x}[\rho (t=\mathrm{1)}]=\sum _{x=-\infty }^{\infty }\,\langle x|\rho (t=\mathrm{1)}|x\rangle =\int \frac{dk}{2\pi }{ {\mathcal L} }_{k}|\varphi \mathrm{(0)}\rangle \langle \varphi \mathrm{(0)}|.$$

The state *ϕ*(0) is the initial state for the coin. The expression of the operator ${ {\mathcal L} }_{k}$ is

23 $${ {\mathcal L} }_{k}=(\begin{array}{cccc}1 & 0 & 0 & 0\\ 0 & 0 & \mathrm{(1}-f)\,\sin \,2k & \cos \,2k\\ 0 & 0 & -\mathrm{(1}-f)\,\cos \,2k & \sin \,2k\\ 0 & 1-f & 0 & 0\end{array}).$$

Here, we use the representation that transform one 2 by 2 matrix to one 4 by 1 column vector, and the coefficient *f* stands for the strength of the noise. After taking the *t* steps evolution of QW, the reduced density matrix for the coin is

24 $${\rho }_{c}(t)=\int \frac{dk}{2\pi }{ {\mathcal L} }_{k}^{t}|\varphi \mathrm{(0)}\rangle \langle \varphi \mathrm{(0)}|.$$

The reduced density matrix for the coin can be interpreted as

25 $${\rho }_{c}(t\to \infty )={\int }_{-\pi }^{\pi }\,\frac{dk}{2\pi }{ {\mathcal L} }_{k}^{t\to \infty }|\varphi \mathrm{(0)}\rangle \langle \varphi \mathrm{(0)}|=(\begin{array}{cccc}1 & 0 & 0 & 0\\ 0 & 0 & 0 & 0\\ 0 & 0 & 0 & 0\\ 0 & 0 & 0 & 0\end{array})\,(\begin{array}{c}{r}_{0}\\ {r}_{1}\\ {r}_{2}\\ {r}_{3}\end{array}).$$

From the derivation above, we find that no matter what the initial state of the coin is, when the coin-decoherence is considered, the reduced density matrix for the coin in the infinite time limit is

26 $${\rho }_{c}(t\to \infty )=\frac{1}{2}(\begin{array}{cc}1 & 0\\ 0 & 1\end{array}).$$

### The populations of the coin in two dimensional QWs with noises

Now, we study the populations of the coin in the two dimensional QW. Firstly, the broken line noise is introduced into the walk. We assume that the broken line noise appears only in the *x*-direction, and four possible evolutions of the two dimensional QW involving decoherence are included. The reduced density matrix for the coin *ρ*_*c*_(*t*) is expressed as

27 $${\rho }_{c}(t)={{\rm{Tr}}}_{x,y}[\rho (t)]=\iint \frac{dk}{2\pi }\frac{dp}{2\pi }{ {\mathcal L} }_{k,p}^{t}|\varphi \mathrm{(0)}\rangle \langle \varphi \mathrm{(0)}|.$$

With the operator ${ {\mathcal L} }_{k,p}$ in the system evolution *ρ*_*c*_(*t*) as

28 $$\begin{array}{cc}{ {\mathcal L} }_{k,p}\tilde{O}\\ \quad =(\begin{array}{ccc}1 & 0 & 0\\ 0 & \mathrm{(1}-2f)\cos \,2p & 2f\mathrm{(1}-f)\,\sin \,k\,\cos \,2p+{f}^{2}\,\sin \,2p\\ 0 & \mathrm{(1}-2f)\sin \,2p & -{\mathrm{(1}-f)}^{2}\,\cos \,2k\,\sin \,2p+{f}^{2}\,\cos \,2p\\ 0 & 0 & {\mathrm{(1}-f)}^{2}\,\sin \,2k\end{array}\begin{array}{c}0\\ {\mathrm{(1}-f)}^{2}\,\sin \,2k\,\sin \,2p+2f\mathrm{(1}-f)\cos \,k\,\cos \,2p\\ 2f\mathrm{(1}-f)\,\cos \,k\,\sin \,2p-{\mathrm{(1}-f)}^{2}\,\cos \,2p\,\sin \,2k\\ {\mathrm{(1}-f)}^{2}\,\cos \,2k+{f}^{2}\end{array}) & (\begin{array}{c}{r}_{0}\\ {r}_{1}\\ {r}_{2}\\ {r}_{3}\end{array})\mathrm{.}\end{array}$$

Following the approach above, by doing the integral, we obtain the density matrix of the coin in the infinite time limit as

29 $${\rho }_{c}(t\to \infty )={\int }_{-\pi }^{\pi }\,{\int }_{-\pi }^{\pi }\,\frac{dk}{2\pi }\frac{dp}{2\pi }{ {\mathcal L} }_{k,p}^{t\to \infty }|\varphi \mathrm{(0)}\rangle \langle \varphi \mathrm{(0)}|=(\begin{array}{cccc}1 & 0 & 0 & 0\\ 0 & 0 & 0 & 0\\ 0 & 0 & 0 & 0\\ 0 & 0 & 0 & 0\end{array})\,(\begin{array}{c}{r}_{0}\\ {r}_{1}\\ {r}_{2}\\ {r}_{3}\end{array}).$$

It means no matter what the initial coin state is, the density matrix for the coin *ρ*_*c*_(*t*) in the infinite time limit is

30 $${\rho }_{c}(t\to \infty )=\frac{1}{2}(\begin{array}{cc}1 & 0\\ 0 & 1\end{array}).$$

Next, we study the populations of the coin in the two dimensional QW with the introduction of the coin-decoherence. In our discussion, we assume that coin-decoherence emerges with the probability *f* before each step of the walk, then the walker moves along the *x*-direction and *y*-direction in sequence. The reduced density matrix *ρ*_*c*_(*t*) for the coin can be obtained by tracing out the degrees of freedom for the position, which is similar as Eq. 27. For this two dimensional QW with coin-decoherence, we can obtain the operator ${ {\mathcal L} }_{k,p}$ associated with the evolution of the coin *ρ*_*c*_(*t*) as

31 $${ {\mathcal L} }_{k,p}\tilde{O}=(\begin{array}{cccc}1 & 0 & 0 & 0\\ 0 & \mathrm{(1}-f)\,\cos \,2p & -\mathrm{(1}-f)\,\cos \,2k\,\sin \,2p & \sin \,2k\,\sin \,2p\\ 0 & \mathrm{(1}-f)\,\sin \,2p & \mathrm{(1}-f)\,\cos \,2k\,\cos \,2p & -\,\sin \,2k\,\cos \,2p\\ 0 & 0 & \mathrm{(1}-f)\,\sin \,2k & \cos \,2k\end{array})\,(\begin{array}{c}{r}_{0}\\ {r}_{1}\\ {r}_{2}\\ {r}_{3}\end{array}),$$

Considering the calculation for the reduced density matrix *ρ*_*c*_(*t*) above, the reduced density matrix of the coin *ρ*_*c*_(*t*) in the infinite time limit can be interpreted as

32 $${\rho }_{c}(t\to \infty )={\int }_{-\pi }^{\pi }\,{\int }_{-\pi }^{\pi }\,\frac{dk}{2\pi }\frac{dp}{2\pi }{ {\mathcal L} }_{k,p}^{t\to \infty }|\varphi \mathrm{(0)}\rangle \langle \varphi \mathrm{(0)}|=(\begin{array}{cccc}1 & 0 & 0 & 0\\ 0 & 0 & 0 & 0\\ 0 & 0 & 0 & 0\\ 0 & 0 & 0 & 0\end{array})\,(\begin{array}{c}{r}_{0}\\ {r}_{1}\\ {r}_{2}\\ {r}_{3}\end{array}).$$

It means no matter what the initial coin state is, when the time approaches infinity, the reduced density matrix for the coin *ρ*_*c*_(*t*) is

33 $${\rho }_{c}(t\to \infty )=\frac{1}{2}(\begin{array}{cc}1 & 0\\ 0 & 1\end{array}).$$
